# Efficacy of radiofrequency and laser thermal ablation in solving thyroid nodule-related symptoms and cosmetic concerns. A systematic review and meta-analysis

**DOI:** 10.1007/s11154-022-09743-8

**Published:** 2022-06-29

**Authors:** Roberto Cesareo, Silvia Egiddi, Anda M. Naciu, Gaia Tabacco, Andrea Leoncini, Nicola Napoli, Andrea Palermo, Pierpaolo Trimboli

**Affiliations:** 1 Center of Metabolic Disease, S.M. Goretti Hospital, Latina, Italy; 2 Unit of Metabolic Bone and Thyroid Disorders, Fondazione Policlinico Universitario Campus Bio-Medico, Rome, Italy; 3 Servizio Di Radiologia E Radiologia Interventistica, Istituto Di Imaging Della Svizzera Italiana (IIMSI), Ente Ospedaliero Cantonale (EOC), Lugano, Switzerland; 4 Servizio Di Endocrinologia E Diabetologia, Ospedale Regionale Di Lugano, Ente Ospedaliero Cantonale (EOC), Lugano, Switzerland; 5 Facoltà Di Scienze Biomediche, Università Della Svizzera Italiana (USI), Lugano, Switzerland

**Keywords:** Thermal ablation, Laser, Radiofrequency, Symptoms, Cosmetic, VAS, Review, Meta-analysis

## Abstract

Several studies have showed good/excellent results of thermal-ablation (TA) to reduce volume of benign thyroid nodule (TN). Nevertheless, no systematic review has reported information about clinical achievements with TA. Being the latter of high interest, this systematic review was undertaken to achieve high evidence about the efficacy of TA in reducing TN-related symptoms and cosmetic concerns. Radiofrequency (RFA) and laser (LA) therapies were considered. A comprehensive literature search of online databases was performed on January 2022 looking for studies reporting clinical results obtained by RFA or LA in terms of VAS (namely, Visual Analogic Scale) and cosmetic concerns. Initially, 318 records were found and 14 were finally included in the meta-analysis. VAS data were available in all RFA studies and the pooled mean reduction was of 3.09 points with significant heterogeneity. Cosmetic score data were available in 11 RFA studies and the pooled mean reduction was of 1.45 with significant heterogeneity. Regarding LA studies, 4 series reported VAS data and the pooled mean reduction was of 2.61 points with significant heterogeneity. The analysis of LA data about cosmetic concerns was not performed due to data paucity. Importantly, heterogeneities were not explained by meta-regression analyses using several covariates (i.e., baseline TN volume, follow-up duration, volume reduction rate). This systematic review showed that clinical data about TN TA efficacy are sparse and affected by high unexplained inconsistency. International societies should give indication about how we should clinically select and evaluate patients undergoing TN TA.

## Introduction

Thyroid nodule (TN) is a largely worldwide diffused pathological entity [1]. Among the huge number of patients with TN only a minority have cancer being the vast majority of TNs benign. Benign TN is generally indolent and does not determine local neck symptoms or cosmetic concerns and is usually referred to clinical and ultrasound (US) long-term follow-up with low likelihood to increase over time [2]. However, in a non-negligible percentage of patients with one or more TNs, they may cause problems, such as symptoms of tracheal and esophageal compression, cosmetic concerns and psychological burden [2]. Although surgery has traditionally been considered as the only therapeutic possibility to remove inconveniences associated to TN, during the last two decades other options have been evaluated and proved to be reliable in this context [3, 4]. In particular, several original papers and some solid meta-analyses have showed good/excellent results of thermal-ablation (TA) [5]. Indeed, the major aim of TA is to delete, or at least reduce, both symptoms and cosmetic discomfort associated to the presence of TN and, then, avoid the more invasive surgery. Currently, following an important number of published data, TA is recognized as a highly effective procedure in reducing thyroid nodules volume [6]. Nevertheless, despite the fact that TA aims to resolve clinical issues, while a lot of studies investigated the amount of TN volume reduction with this therapy, to the best of our knowledge no systematic review/meta-analysis have been conceived to obtain solid information about clinical achievements with TA.

Generally, since a thyroid specific scale does not exist, the authors of TA articles reported clinical results of TA according to visual analogic scale (VAS). VAS allows a visual representation of pain and represents one frequently used measure in several adult populations [7], even if its interpretation is sometimes straightforward [7–13]. Alternatively to VAS, other scales, such as that proposed to evaluate the nodule-induced symptoms after percutaneous ethanol injection (PEI) [14], were used. Furthermore, TN TA efficacy have also been evaluated in terms of aesthetic improvement. A cosmetic scoring system was firstly described in 2008 [15] and several subsequent studies have used that to evaluate the effectiveness of TA in TN patients. From the clinical standpoint, since these scoring systems were no initially conceived for thyroid use and they were not actually validated for this use, we should ask ourselves whether they are appropriate to the case of TN TA, and, of course, whether TA is effective to improve symptoms and cosmetic concerns as it is in terms of volumetric reduction of TN.

This systematic review was undertaken to achieve high evidence about the efficacy of TA in reducing or deleting the TN-related symptoms and cosmetic concerns. Accordingly, the present study aimed to evaluate 1) how the TN-related symptoms are deleted or improved according to VAS, and 2) how cosmetic concerns are deleted or solved according to specific scale. For the present study, two most diffused TA procedures, such as radiofrequency (RFA) and laser (LA), were considered.

## Methods

### Conduction of review

The systematic review was performed according to MOOSE (Meta-analysis Of Observational Studies in Epidemiology) [16]

### Search strategy

A five-step search strategy was planned; 1) sentinel studies were searched in PubMed; 2) keywords and MeSH terms were identified; 3) PubMed and Cochrane databases were searched; 4) studies reporting efficacy of RFA or LA in terms of both cosmetic and symptoms were detected; 5) references of included studies were screened for additional papers. Studies with overlapping data and studies including less than 10 cases were excluded. The last search was performed on January 21st, 2022. Articles in English language were always included while those in other languages were translated, when appropriate. No publication year restriction was applied. Two investigators (SE, AMN) independently and in duplicate searched papers, screened titles and abstracts, reviewed full-texts and selected articles for inclusion.

### Data extraction

Following information was independently extracted by two authors (SE, AMN) from the main paper and its supplementary files: 1) general study information (authors, year of publication, country, study type and design), 2) number of patients and nodules, 3) mean and/or median with standard deviation (SD) and error (SE), respectively, of VAS or other scales at both baseline and end of the study, 4) mean (and SD) and/or median (and SE) nodules’ baseline volume, 5) mean (and SD) and/or median (and SE) nodules’ volume reduction rate (VRR) at the end of the study. Data were cross-checked between the two authors and discrepancies were mutually discussed with the other authors.

### Study quality assessment

Two authors (SE, AMN) performed the risk of bias assessment according to National Heart, Lung, and Blood Institute Quality Assessment Tool for Observational Studies [17].

### Study outcome measures

Measures included in the present study were VAS and cosmetic score, being they the most diffused ones to evaluate the efficacy of TN TA from the clinical point of view. In addition, since VRR is the most diffused measure to evaluate the radiological efficacy of TA in TN, it was here used as the most important covariate to explore the results of VAS and cosmetic score. As above mentioned, the large majority of studies aimed at evaluating TA efficacy in terms of symptomatology have reported clinical results according to VAS. This was initially conceived to evaluate pain in adults and consists in a visual representation of the amplitude of pain felt by the patient by a predetermined 10-cm line where the extremes correspond to “no pain” (left extreme) and "severe pain" (right extreme). Patient is asked to draw a sign on the line that represents the level of pain he feels. Then, VAS score is calculated in millimeters, by measuring with a ruler the distance between the extreme corresponding to the minimum intensity and the mark placed by the patient. On the basis of several studies, cut-off values have been suggested as follows: 0 to 4 mm: “no pain”; 5 to 44 mm: "mild pain"; 45 to 74 mm: "moderate pain"; 75 to 100 mm: "severe pain" [7, 8]. With these features, VAS is a valid and reliable estimate of acute and chronic pain intensity [9–12]. In fact, pain rating scales have a fundamental place in clinical practice even if the interpretation of pain scores is not straightforward [13]. Other scales were proposed as alternative to VAS (see below) but they were rarely used. Anyway, neither VAS nor other scales have never been actually validated for TN TA by large prospective trials. The efficacy of TA in TN patients has also been evaluated in terms of aesthetic improvement. As above mentioned, the cosmetic scoring system firstly described in 2008 has been the most diffusely used in this context [15]. This scale includes the following 4 classes: 1-no palpable mass, 2-no cosmetic problem but palpable mass, 3-cosmetic problem on swallowing only, 4-a readily detected cosmetic problem. The cosmetic score is assessed by TA operators to evaluate whether TA is able to improve the aesthetic discomfort associated to TN. Interestingly, the first article using this scoring system did not report a specific literature reference about it [15]. Finally, almost all studies about TN TA have evaluated the efficacy of the therapy in terms of TN shrinkage. In particular, VRR was the major measure in this context and it was calculated according to the following formula: (baseline volume – final volume) × 100 / baseline volume. VRR is expressed as a percentage and it is generally considered that the higher the VRR the higher the TA efficacy.

### Statistical analysis

Studies' characteristics were summarized. When at least four studies are available to be pooled, a proportion meta-analysis was performed to calculate the mean difference of VAS or other scales to estimate the efficacy of TA in terms of cosmetic concerns or symptoms. A random-effect model was used. Pooled data were presented with 95% confidence intervals (95% CI). Heterogeneity was assessed by using I_2_ with a value ≥ 50% meaning high heterogeneity. Meta-regression or subgroup analyses were performed to explore heterogeneity when it is found. A p < 0.05 was regarded as significant. Statistical analyses were performed using OpenMeta[Analyst] (open-source software developed by the Center for Evidence Synthesis in Health, Brown University, Providence, RI, USA).

## Results

### Studies retrieved

**Fig. 1:**
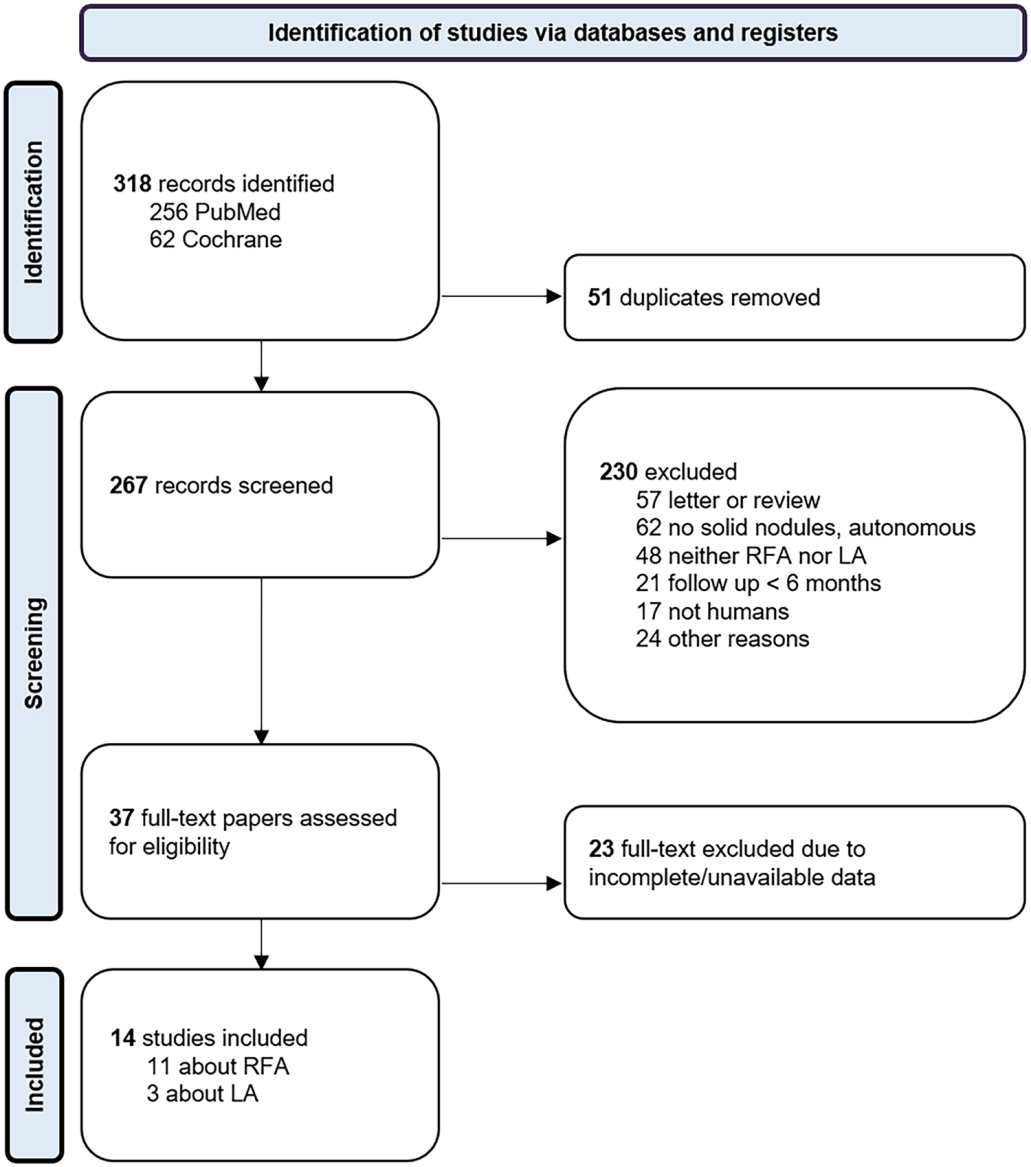
Flow of records of the systematic review

A total of 318 records were initially found according to the above strategy. After reviewing their title and abstract 267 papers were initially selected and evaluated for the inclusion, 37 studies were initially included (30 about RFA and 7 about LA), and 14 studies were finally included in the meta-analysis (Fig. 1, Table 1) [18–31].

**Table 1:** Characteristics of studies included in the meta-analysis

Authors	Journal	Year of publication	Mean patient’s age (SD)	TA	Sample size (TN number)	Symptom score scale	Cosmetic score scale	Follow-up after TA (months)
Baek et al. [18]	Am J Roentgenol	2010	47.5 (9)	RFA	15	0–10	1–4	6
Huh et al. [19]	Radiology	2012	37.5 (11.5)	RFA	15	0–10	1–4	6
Cesareo et al. [20]	J Clin Endocrinol Metab	2015	56 (14)	RFA	42	0–10	1–4	6
Deandrea et al. [21]	Thyroid	2015	NA	RFA	40	0–10	1–4	6
Døssing et al. [29]	Thyroid	2006	NA	LA	30	0–10	0–10	6
Cesareo et al. [22]	Arch Endocrinol Metab	2017	57.7 (14)	RFA	48	0–10	1–4	12
Feroci et al. [23]	Surg Innov	2020	57.2 (17.1)	RFA	32	0–10	1–4	12
Jeong et al. [24]	Ultrasonography	2022	54.9 (12.9)	RFA	55	0–10	1–4	12
Ha et al. [25]	Endocrinol Metab	2019	43.8 (12.3)	RFA	16	0–10	1–4	12
Shi et al. [30]	Front Endocrinol	2019	44.5 (21.4)	LA	180	0–10	1–4	12
Negro et al. [31]	Korean J Radiol	2020	54 (45–66)*	LA	104	0–10	1–4	12
Valcavi et al. [26]	Endocrine Pract	2015	54.9 (14.3)	RFA	40	0–10	0–10	24
Guang et al. [27]	BMC Cancer	2019	47.6 (11.3)	RFA	194	0–10	1–4	24
Yan et al. [28]	Int J Hyperthermia	2020	46.56 (11.8)	RFA	206	0–10	1–4	24

Articles are ordered according to the duration of follow-up after TA. *data of this paper are expressed as median and interquartile ranges

### Study quality assessment

Quality assessment is detailed in Table 2. Overall, the risk of bias was low. However, studies were at high risk of bias with respect to sample size and authors did not report the analysis of power or sample.

**Table 2:** Risk of bias summary: review of authors’ judgements about each risk of bias item for each included observational study

First author, year	**1**	**2**	**3**	**4**	**5**	**6**	**7**	**8**	**9**	**10**	**11**	**Total**
Baek, 2010	Yes	Yes	Yes	Yes	No	Yes	Yes	No	No	Yes	Yes	8/11
Huh, 2012	Yes	Yes	Yes	No	No	Yes	Yes	No	No	Yes	Yes	7/11
Cesareo, 2015	Yes	Yes	Yes	Yes	No	Yes	Yes	No	No	Yes	Yes	8/11
Deandrea, 2015	Yes	Yes	Yes	Yes	No	Yes	Yes	No	No	Yes	Yes	8/11
Døssing, 2006	Yes	Yes	Yes	Yes	No	Yes	Yes	No	No	Yes	Yes	8/11
Cesareo, 2017	Yes	Yes	Yes	No	No	Yes	Yes	No	No	Yes	Yes	7/11
Feroci, 2020	Yes	Yes	Yes	Yes	No	Yes	Yes	No	No	Yes	Yes	8/11
Jeong, 2022	Yes	Yes	Yes	No	No	Yes	Yes	No	No	Yes	Yes	7/11
Ha, 2019	Yes	Yes	Yes	Yes	No	Yes	Yes	No	No	Yes	Yes	8/11
Shi, 2019	Yes	Yes	Yes	Yes	No	Yes	Yes	No	No	Yes	Yes	8/11
Negro, 2020	Yes	Yes	Yes	No	No	Yes	Yes	No	No	Yes	Yes	7/11
Valcavi, 2015	Yes	Yes	Yes	Yes	No	Yes	Yes	No	No	Yes	Yes	8/11
Guang, 2019	Yes	Yes	Yes	No	No	Yes	Yes	No	No	Yes	Yes	7/11
Yan, 2020	Yes	Yes	Yes	No	No	Yes	Yes	No	No	Yes	Yes	7/11

Questions:

1. Was the study question or objective clearly stated?

2. Were eligibility/selection criteria for the study population prespecified and clearly described?

3. Were the participants in the study representative of those who would be eligible for the test/service/intervention in the general or clinical population of interest?

4. Were all eligible participants that met the prespecified entry criteria enrolled?

5. Was the sample size sufficiently large to provide confidence in the findings? Did the authors present their reasons for selecting or recruiting the number of individuals included or analyzed?

6. Was the test/service/intervention clearly described and delivered consistently across the study population?

7. Were the outcome measures prespecified, clearly defined, valid, reliable, and assessed consistently across all study participants?

8. Were the people assessing the outcomes blinded to the participants' exposures/interventions?

9. Was the loss to follow-up after baseline 20% or less? Were those lost to follow-up accounted for in the analysis?

10. Did the statistical methods examine changes in outcome measures from before to after the intervention? Were statistical tests done that provided p values for the pre-to-post changes?

11. Were outcome measures of interest taken multiple times before the intervention and multiple times after the intervention (i.e., did they use an interrupted time-series design)?

### Qualitative analysis (systematic review)

The seventeen articles were published between 2002 and 2022. Most studies were conducted using RFA and only a minor part used LA. The authors were from Europe in 7 cases and from Asia in the other 7. The sample size ranged from 15 to 206 TNs. The same 10-point VAS scale was used in all studies. Cosmetic score was evaluated by a 4-point scale in all studies but three. The follow-up duration after TA ranged from 6 to 24 months.

### Quantitative analysis (meta-analysis) of RFA data: symptom score

**Fig. 2:**
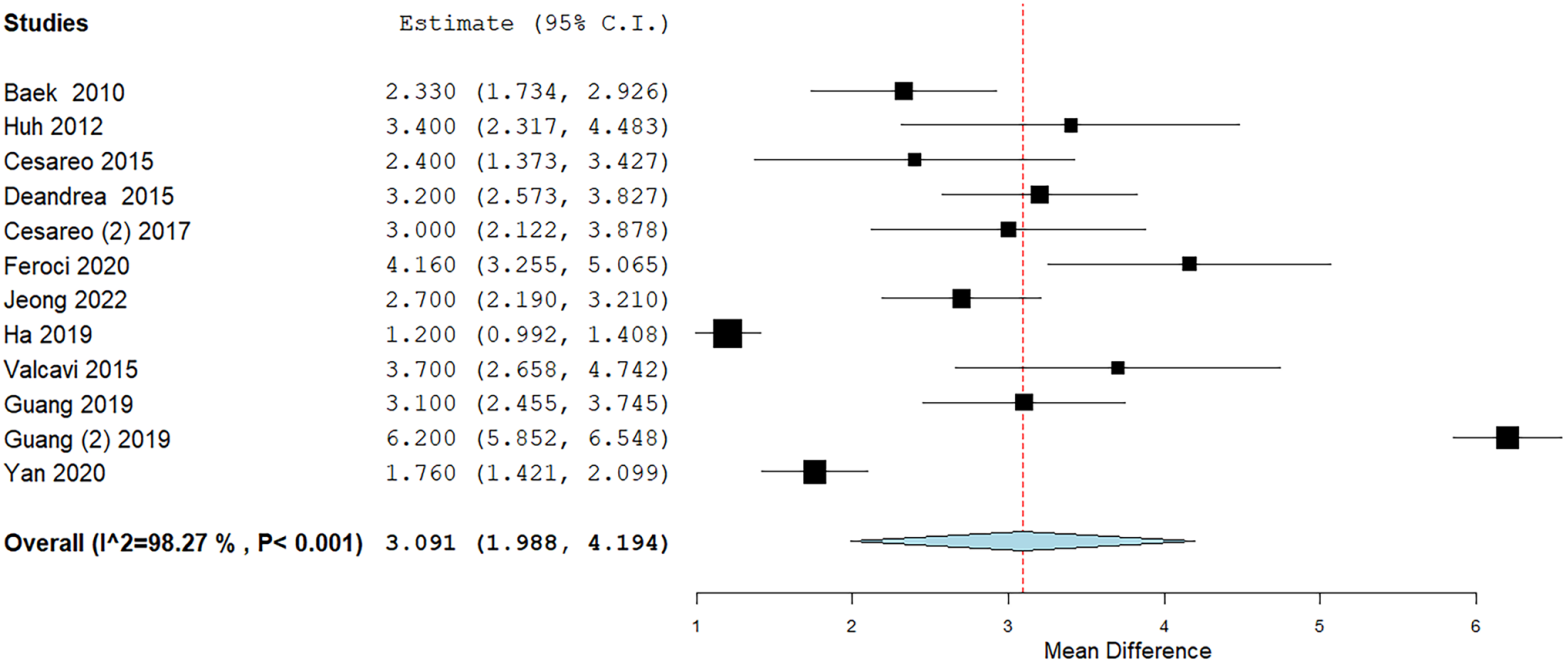
VAS variation after RFA. Blu diamond represents the pooled difference and its wideness is correlated to 95%CI. Square indicates the study sample size and line indicates 95% CI

**Fig. 3:**
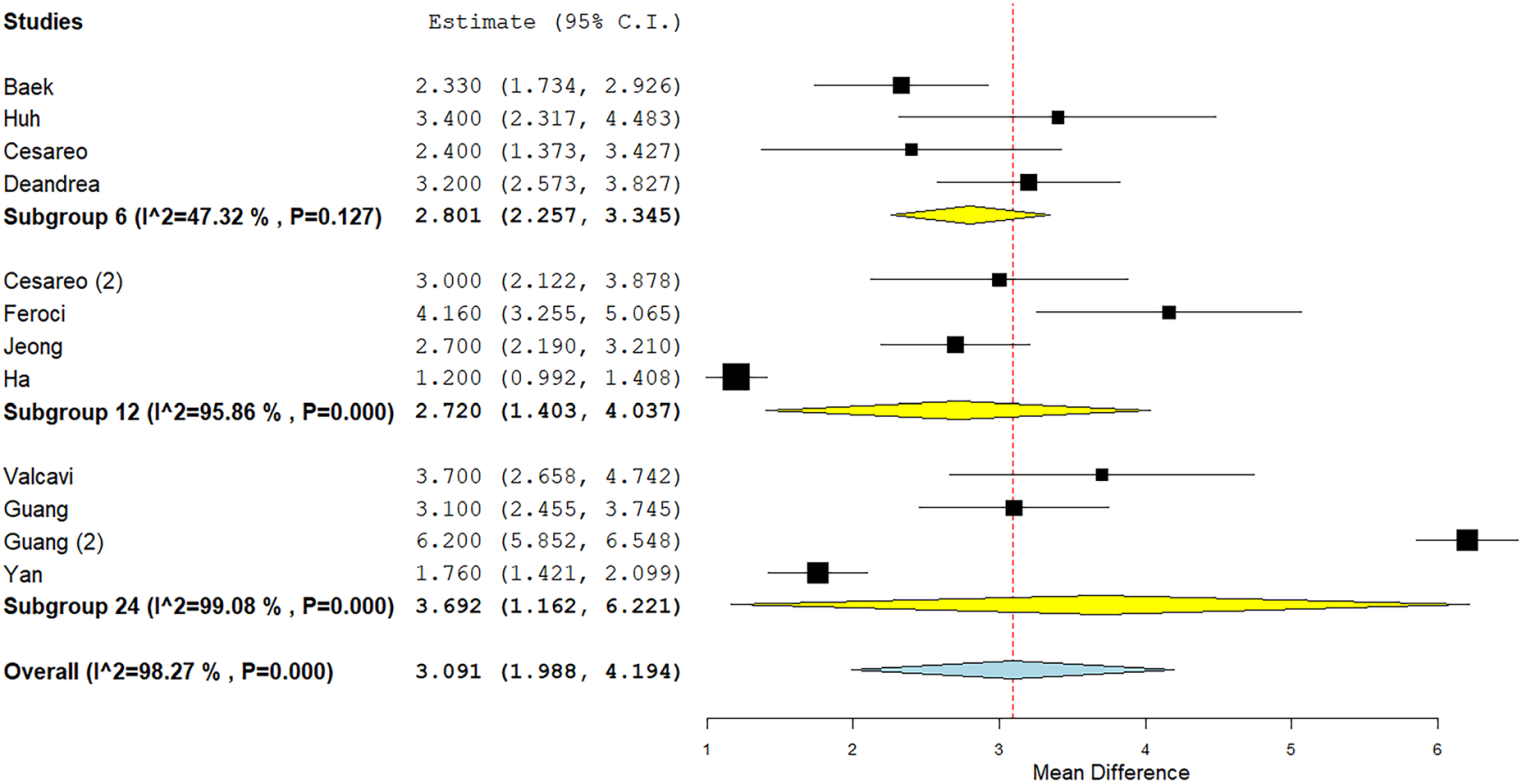
VAS variation after RFA according to the duration of follow-up after TA. Diamond (yellow for subgroup, blue for the pooled group) indicates the pooled difference and its wideness indicates 95% CI. Square indicates the study sample size and line indicates 95% CI

**Fig. 4:**
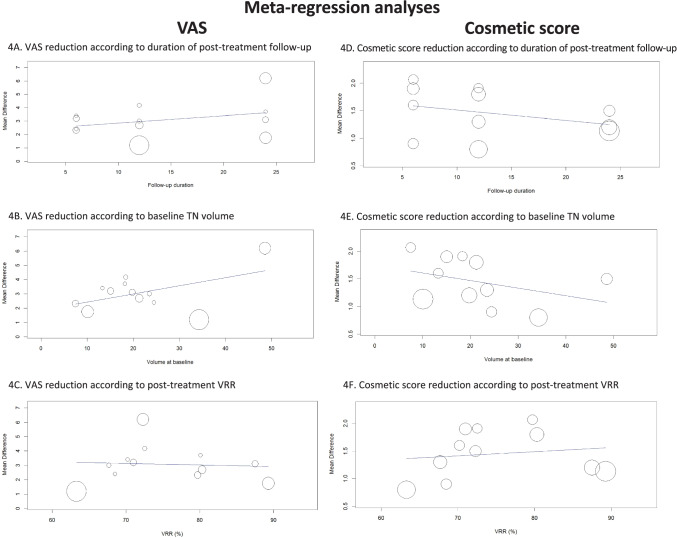
Meta-regression analyses to explore heterogeneity in variation of VAS and cosmetic concerns score after RFA. Any circle identifies a study and its size is according to the study weight. TN, thyroid nodule; VRR, volume reduction ratio

VAS data were available in all the included studies. When pooling all findings, a mean reduction by 3.09 points were observed with significant heterogeneity (Fig. 2). To explore the heterogeneity multiple sub-analyses were performed. The VAS results were evaluated by a subgroup-analysis dividing the studies according to the duration of post-therapy follow-up (Fig. 3) and with a specific meta-regression (Fig. 4A) but it remained unsolved (p = 0.25). In addition, the heterogeneity could not be explained when the baseline TN volume was used as covariate (Fig. 4B) even if a trend was observed, i.e., the larger the basal volume the larger the VAS reduction (p = 0.056).

Finally, heterogeneity remained unsolved (p = 0.82) when VRR was used as covariate (Fig. 4C).

### Quantitative analysis (meta-analysis) of RFA data: cosmetic score

**Fig. 5:**
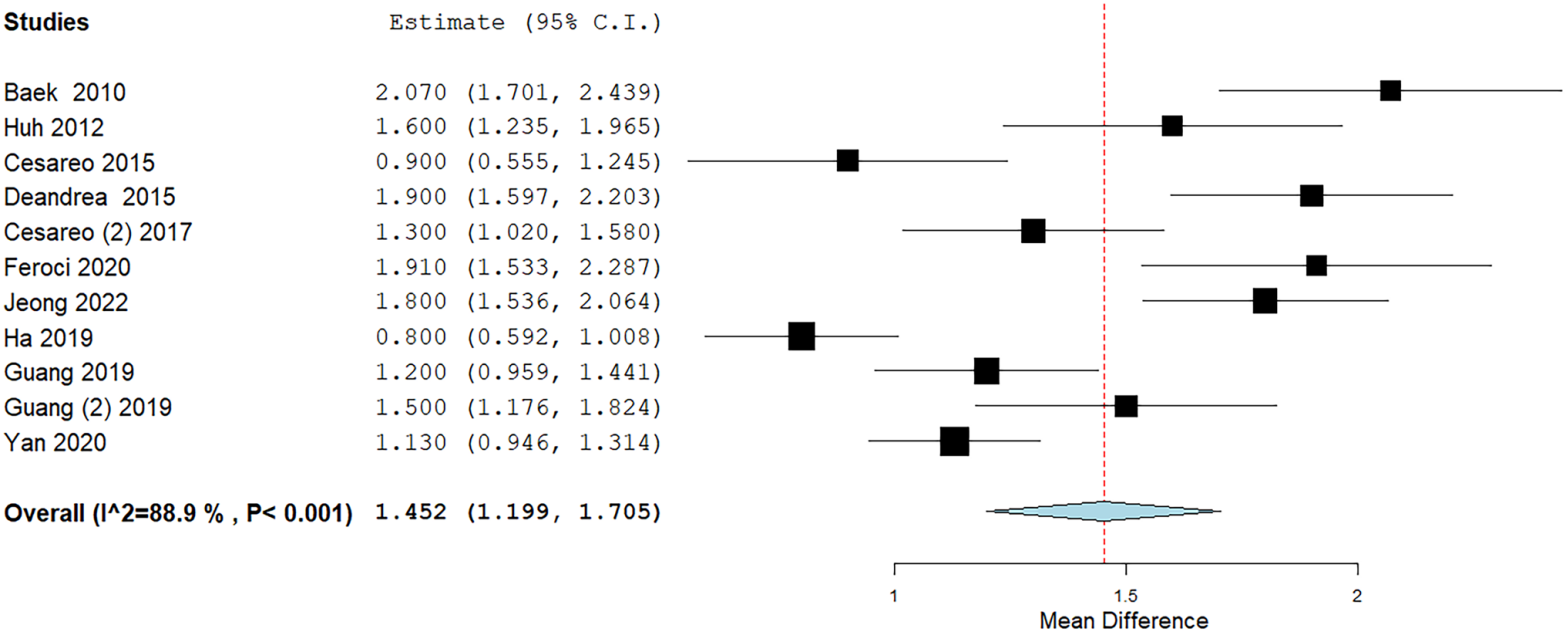
Cosmetic score variation after RFA. Blu diamond represents the pooled difference and its wideness is correlated to 95%CI. Square indicates the study sample size and line indicates 95% CI

**Fig. 6:**
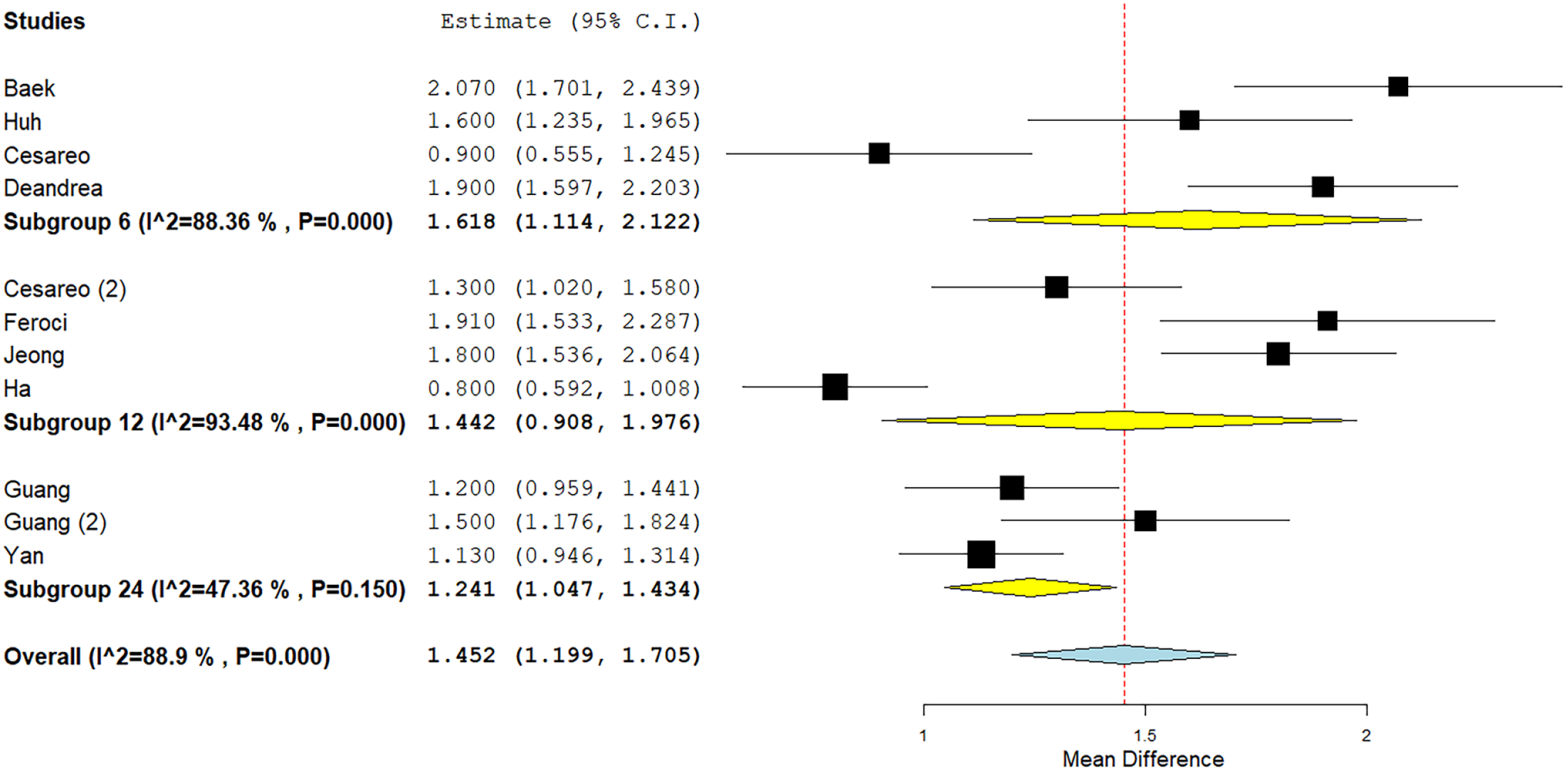
Cosmetic score variation after RFA according to the duration of follow-up after TA. Diamond (yellow for subgroup, blue for the pooled group) indicates the pooled difference and its wideness indicates 95% CI. Square indicates the study sample size and line indicates 95% CI

The TA result in terms of cosmetic score could be analyzed by pooling those 11 studies using a 10-point scale (it was excluded only the paper by Valcavi et al. [26] that reported findings in another scale). Mean reduction of value was 1.45 with significant heterogeneity (Fig. 5). The heterogeneity was explored considering the duration of post-treatment follow-up but, even if a trend should be observed in a sub-group analysis (i.e., the shorter the follow-up the higher the variation of score), heterogeneity remained unsolved (Fig. 6), also when a specific meta-regression was performed (p = 0.24) (Fig. 4D). In addition, to better explore the heterogeneity, a meta-regression (Fig. 4E) using mean baseline volume of TN as covariate but heterogeneity was unchanged again (p = 0.18). Finally, a meta-regression using VRR as covariate did not explain (p = 0.62) the heterogeneity found among the studies (Fig. 4F).

### Quantitative analysis (meta-analysis) of LA data: symptom score

**Fig. 7:**
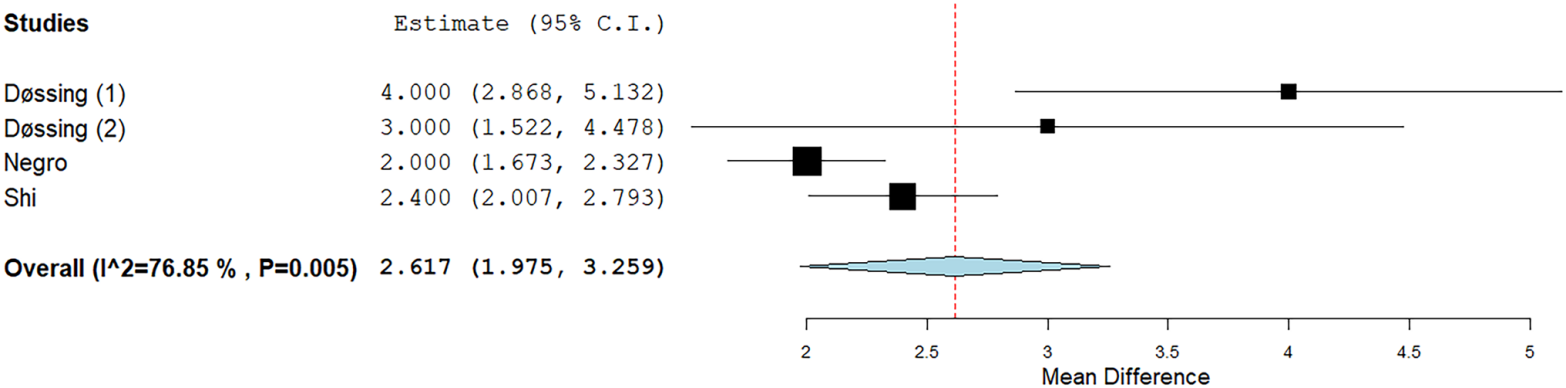
VAS variation after LA. Blu diamond represents the pooled difference and its wideness is correlated to 95%CI. Square indicates the study sample size and line indicates 95% CI

VAS data were available in four series. When pooling these findings, a mean reduction by 2.61 points were observed with significant heterogeneity (Fig. 7). Due to the paucity of data, heterogeneity was not explored.

### Quantitative analysis (meta-analysis) of LA data: cosmetic score

This analysis was not performed due to the sparse available data and the heterogeneous scoring systems used in the studies (Table 2).

## Discussion

Thermal ablation was proven as reliable alternative to surgery due to its effectiveness in reducing the volume of thyroid nodules and this is supported by several prospective randomized trials and meta-analyses [6]. Therefore, international societies have developed guidelines and recommendations regarding the correct use of these procedures, especially those most diffused such as RFA and LA. The most recent guidelines [32] converge on the point that these treatments should be done only in symptomatic patients, that is, those suffering from compression and/or cosmetic problems specifically associated to the presence of TN. However, most papers that validated the use of these procedures really focused on their effectiveness in terms of volumetric reduction of TN (i.e., they used only VRR as major endpoint). In these studies, the improvement of symptoms and cosmetic scores with TA, when described, was reported as secondary endpoint and the evaluation of these aspects, particularly symptom concerns, has been done using non-validated scales.

Before discussing the different reported findings of the meta-analyses, it’s important to shed light on the use of these scales to evaluate TN-related symptoms and cosmetic concerns. To the best of our knowledge, VAS scale was validated as a ratio scale measures for both chronic and experimental pain in 1983 [33]. For the first time, in 2002, Dossing et al. adopted VAS to assess the effects of LA on nodule-related symptoms in patients with solid cold thyroid nodules [34]. Later, in 2008, Deandrea et al. used VAS scale to analyze the degree of compressive symptoms in patients with solid benign thyroid nodules treated with RFA [35]. Successively, all the main studies aimed to investigate the clinical impact of TN TA adopted VAS. It is worth to be mentioned that other scales were proposed as alternative to VAS in the field of TN TA. In example, Zingrillo and colleagues tested in 1998 the clinical efficacy of PEI in cold benign thyroid nodules by the use of a different scale that consisted of a score ranging from 0 to 6 [14]. Three symptoms, i.e., pressure in the neck, dysphagia and aesthetic complaint, were included in this scale and they were scored separately as follows: 0-absent, 1-moderate, and 2-severe. A few authors adopted the same scale, such as Spiezia et al. in his prospective study aiming to investigate the long-term effectiveness of RFA in controlling compressive symptoms due to solid or mainly solid benign thyroid nodules [36]. Since data about these alternative scales were sporadic, a meta-analysis was not feasible. About the nodule related cosmetic concerns, the first author who adopted the 4-point cosmetic scale was Jin et al. in 2008. The purpose of his study was to evaluate the efficacy and safety of one-step ethanol ablation in the management of viscous cystic thyroid nodules [15]. In 2010 Baek and colleagues applied for the first time this scale to test the clinical efficacy of RFA on benign predominantly solid thyroid nodules [18]. As a consequence of borrowing inappropriate tools to evaluate these clinical outcomes, while several meta-analyses showing a significant VRR over 6 to 12-month post-treatment follow-up, no evidence-based data about symptom and cosmetic score have been published indeed. Following these premises, the present systematic review was conceived to achieve solid information about the results of RFA and LA in deleting/reducing symptoms and cosmetic concerns associated to TN. Then, the herein reported findings should be fully discussed.

First, the number of papers reporting data about the improvement of symptom and cosmetic scores with TA seem to be scarce, in particular for LA. We should ask ourselves whether the latter is due to the less important diffusion of LA or whether LA has reduced appeal during the last years as showed by the fact that the number of studies about TN LA has been recently lowered than that abouth RFA [5]. Anyway, we must take into account that only a minority of papers about TN TA was focused on the clinical impact of this therapy.

Second, the pooled findings about improvement of symptom and cosmetic score are highly heterogeneous and the latter cannot be explained despite several meta-regressions are performed using RFA data. What is particularly striking is that there appears to be no correlation between VRR and improved clinical data. This result merits a careful re-evaluation of the scoring systems used to analyze the clinical efficacy of thyroid TA. In addition, these findings advice for further prospective studies mainly aimed to evaluate the clinical results rather than the volumetric ones. Also, data about LA derived from a small number of studies and two series were published by the same authors.

Third, the pooled mean at baseline of VAS, a 10-point scale, was 4.0 in RFA studies and 4.2 in LA studies. This data should intrinsically mean that the indication for TA was mild-to-moderate. We must ask again ourselves whether VAS is an appropriate tool in the context of benign TN.

Basically, present findings show that the generally used scales to evaluate the improvement of symptoms and cosmetic concerns after TA are not correlated with the results in terms of volumetric shrinkage of TN. Intrinsically, this result should prompt to revise those indicators we generally use to treat our patients with TA. From a clinical point of view there is a need of a well-structured and thyroid nodule-oriented tool to evaluate symptoms and cosmetic concerns correlated to the presence of a TN. The latter should be developed by a well-designed multicenter trial involving large-volume centers having a large experience in TN TA.

Limitations and strengths of the present meta-analysis should be discussed. The included published data were from observational and mainly retrospective series. Papers reporting data of symptom and cosmetic score in median were sparse and they could not be included in the systematic review. Some papers included in the present meta-analysis were published about twenty years ago while some other were very recent. In some studies, the post-treatment follow-up was quite short. Generally, both symptoms and cosmetics concerns were secondary endpoints, being the primary one the volumetric change of TN after TA. In some studies, the scale used to evaluate the improvement of VAS and cosmetic concerns was different from the other ones and these studies were excluded from the pooled analysis.

## Conclusions

According to the study aims, we can conclude that 1) sparse data about TN TA efficacy in terms of symptom and cosmetic scale have been published, particularly regarding LA; 2) symptom and cosmetic scores improve after RFA with high inconsistency among studies, being this unexplained. Based on these findings, international societies should give clear indication about how we have to select patients to be treated by TA and how we can evaluate the clinical result with TA. Basically, current international recommendations to treat only symptomatic patients are not supported by the literature.

## Data Availability

The datasets generated during and/or analyzed during the current study are not publicly available but are available from the corresponding author on reasonable request.

## List of abbreviations

LA: Laser ablation
MOOSE: Meta-analysis Of Observational Studies in Epidemiology
PEI: Percutaneous ethanol injection
RFA: Radiofrequency ablation
SD: Standard deviation
SE: Standard error
TA: Thermal-ablation
TN: Thyroid nodule
US: Ultrasound
VAS: Visual Analogic Scale
VRR: Volume reduction rate
